# Value of CT angiography in reducing the risk of hemorrhage associated with mini-percutaneous nephrolithotomy

**DOI:** 10.1590/S1677-5538.IBJU.2014.0306

**Published:** 2015

**Authors:** Xiang-Jun Meng, Qi-Wu Mi, Tao Hu, Wei-De Zhong

**Affiliations:** 1 Southern Medical University, Guangzhou, Guangdong, P.R. China; 2 Department of Urology, Dongguan people's Hospital, Dongguan 523059, Guangdong, P.R. China; 3 Department of Radiology, Dongguan people's Hospital, Dongguan 523059, Guangdong, P.R. China; 4 Department of Urology, Guangzhou first people's Hospital 510180, Guangzhou, Guangdong, P.R. China

**Keywords:** Nephrostomy, Percutaneous, Urinary Tract, Calculi, Tomography, X-Ray Computed, Angiography

## Abstract

**Purpose:**

To evaluate the clinical value of computed tomography angiography (CTA) in reducing the risk of hemorrhage associated with mini-percutaneous nephrolithotomy (PCNL).

**Materials and Methods:**

A total of 158 patients with renal or ureter stones who had undergone mini-percutaneous nephrolithotomy were retrospectively enrolled into this study from May of 2011 to April of 2014. Group 1 (65 patients) underwent computed tomography angiography, and Group 2 (93 patients) underwent non-contrast CT. The clinical characteristics of the patients and hemorrhagic complications were recorded. The hematologic complications (transfusion rate, and preoperative and postoperative hemoglobin values) were assessed.

**Results:**

There were no statistically significant differences in age, body mass index(BMI), stone diameter, operative time, stone-free rate, and hospital stay between the 2 groups. In group 2, 1 patient (1.1%) developed a renal arteriovenous fistula and was treated with embolus therapy. In addition, Group 2 showed significantly drop in hemoglobin (3.6 g/dL vs. 2.4 g/dL, respectively; P <0.001) and more transfusions (9.7% vs. 1.5%, respectively; P <0.05) compared with Group 1.

**Conclusion:**

The study showed that patients who underwent computed tomography angiography prior to percutaneous nephrolithotomy had lower drop of hemoglobin and needed less transfusions. These findings may suggest that the use of computed tomography angiography may reduce the risk of bleeding during percutaneous nephrolithotomy.

## INTRODUCTION

Percutaneous nephrolithotomy has become an effective and valued procedure in the treatment of kidney and ureter stones. Although PCNL is a minimally invasive technique, it carries the potential risk of complications.

The complication rates associated with PCNL range from 29% to 83% (1–3). In the percutaneous nephrolithotomy Global Study analysis, the bleeding complication rate and transfusion rate were 9.4% and 7.0%, respectively (4). Bleeding is a serious complication which is usually caused by renal vascular lesions. These lesions must be controlled by any number of maneuvers, such as blood transfusions, deployment of larger nephrostomy catheters, or renal embolus therapy. Bleeding can lead to longer hospital stays and injure patients. Therefore, an important question is how to reduce the hemorrhage associated with percutaneous nephrolithotomy.

We report our experience in reducing the risk of hemorrhage associated with mini-percutaneous nephrolithotomy with the use of pre-procedural computed tomography angiography.

## MATERIALS AND METHODS

**Figure 1:**
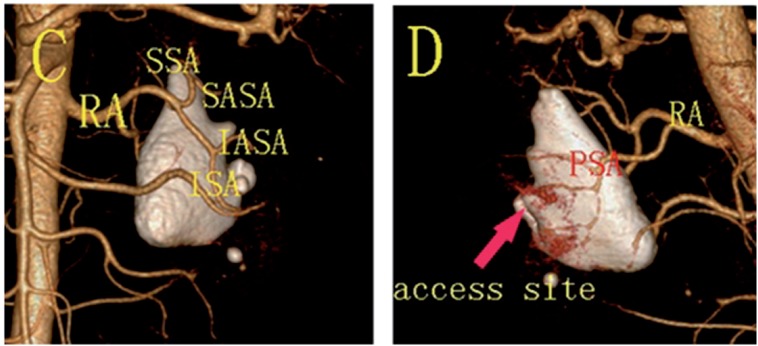
Computed tomography angiography.

**Figure 2:**
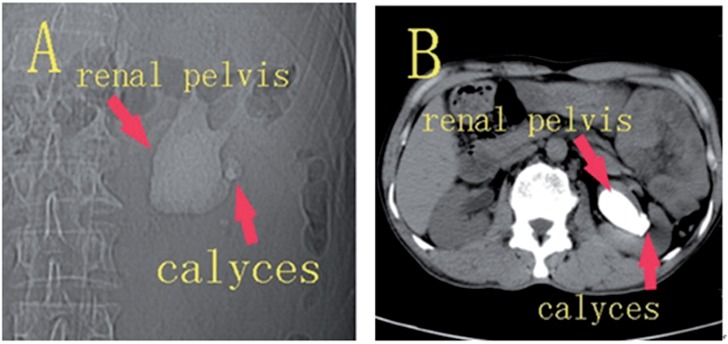
Non-contrast computed tomography.

From May of 2011 to April of 2014, 158 patients with renal or ureter stones were retrospectively enrolled into 2 groups, and they underwent a mini-percutaneous nephrolithotomy. In Group 1 (65 patients), the patients underwent a preoperative computed tomography angiography (Figure-1), and in Group 2 (93 patients), the patients underwent non-contrast CT (Figure-2). Surgery was performed by a surgeon with 10 years of experience after the computed tomography angiography examination. Patients were excluded if they had comorbidities which might affect bleeding risk such as diabetes, hypertension, high serum creatinine (Cr >200 umol/L). During follow-up, that ranged from 1 to 19 months, 1 case was lost to follow-up.

The study protocol was approved by the institutional review board, the patients in this manuscript has given written informed consent to publish these case details.

### 

#### Computed tomography angiography Protocol

A multidetector spiral CT (Brilliance iCT, Philips Medical Systems, Cleveland, Ohio) was used (65 patients), and CTA was performed with the following parameters: standard modality, gantry rotation 0.4s, pitch 0.915, KV 120, mAs 300, collimation 128*0.625, matrix 512*512, thickness 0.9mm, increment-0.45mm, and Dose Length Product(DLP) 802.3mGy*cm.

A contrast agent (Ultravist, 370mgI/mL was used via the CT high-pressure syringe, with a moderate injection rate(4~5 mL/s) and dose(1.0~1.5 mL/ kg). Arterial, venous, and urographic phases were acquired after a bolus tracking test.

Images were post-processed on an independent workstation (Extended Brilliance Workspace V4.5, Philips Medical Systems, Cleveland, Ohio) to obtain multiplanar reconstructions (MPRs), maximum intensity projections (MIPs), as well as surface and volume renderings (Figure-1).

The computed tomography angiography was reviewed carefully by two radiologistes individually who were blinded to this study. Disagreements were resolved by two radiologists in a final consensus reading.

#### Surgical Protocol

In Group 1, before the procedure, the CT angiography was analyzed carefully by two radiologist who obtained renal vasculature map. The selection of an access site far away from larger vessels was performed by the radiologist, and the urologist performed the puncture in view of the selected access site(s). In Group 2, the urologist used non-contrast CT to select access site(s) and perform the puncture.

All patients underwent the placement of an ipsilateral ureteral catheter under general anesthesia and were turned to a full prone position. In Group 1, based on the computed tomography angiography, access calyces were identified. In Group 2, renal access was obtained basing on non-contrast CT. The puncture was performed with fluoroscopic guidance by the urologist in view of the distribution of the stones. Dilation was done with serial manual dilators up to a size of 20-Fr, and a 20-Fr Amplatz sheath (COOK medical inc., Bloomington, USA) was positioned in the target renal calyx. Wolf nephroscope (11.5Fr) was used in the mini-percutaneous nephrolithotomy, and the stones were fragmented with an pneumatic lithotripsy device (Hawk medical inc., Shenzhen, China).

No residual fragments were defined as stone-free. The outcome was confirmed by performing a B-ultrasound or abdominal plain film examination on the third postoperative day.

Hemoglobin level was measured on preoperative day and on the first postoperative day, or when severe acute bleeding occurred. A blood transfusion was required when hemoglobin level ≤7g/dL or a progressive drop in the hemoglobin level was observed (≤10g/dL).

### Statistical analysis

The statistical analysis was performed using SPSS 17.0 (SPSS, Chicago, IL, USA). The data are reported as the mean plus or minus the standard deviation (SD) or as the median and range. Continuous variables were compared using the Wilcoxon rank sum test, and nominal variables were analyzed using the Pearson chi square test. A P value <0.05 was considered statistically significant.

## RESULTS

The patient demographics and stone characteristics are reported in Table-1. The two Groups were comparable in terms of age, the male to female ratio, BMI(body mass index), side and stone diameter. There were no significant differences in the operation time, stone-free rate hospital stay and access tract number (number of renal calix punctured per patient) between the two Groups (Table-2).

**Table 1:** Patient demographics and stone characteristics\operative details and outcomes in the two Groups.

Variable	Group 1	Group 2	P value
No. of patients (n)	65	93	NA
Mean age, years mean±SD (range)	40.6±12.5(18~75)	40.7±10.0(20~73)	0.943
Male:female (n)	34:31	43:50	0.45
BMI, kg/m^2^, mean±SD (range)	22.5±1.5(19.0~25.3)	22.6±1.3(19.2~24.9)	0.638
Right/left side (n)	30:35	48:45	0.50
Mean max stone diameter (cm),mean±SD (range)	3.6±0.8(2.1~6.0)	3.4±0.6(1.9~6.4)	0.068
Multiple stones (n)	38	56	0.83

**Table 2:** Operative details and outcomes in the two groups.

Variable	Group 1	Group 2	P value
Operation time(min) mean±SD	75.5±28.4	77.6±23.0	0.604
Stone-free rate(%)	87.7	88.2	0.60
Mean hospital stay (d) (SD)	8.1±1.7	8.3±1.5	0.279
Access number	1.2±0.5	1.3±0.6	0.241

Computed tomography angiography was performed in Group 1, the renal main trunks and vessel branches were displayed accurately. Therefore, the computed tomography angiography provided a good map of the renal vasculature, and had an advantage over non-contrast CT in optimizing the placement of nephrostomy (Figures 1 and 2).

Three cases in Group 1 suffered urinary sepsis (fever<39°C) and two cases in Group 2. A prolonged urinary leak from the flank was observed in one case in Group 1, which spontaneously stopped without requiring a double J stent positioning.

A greater drop in the hemoglobin level (P<0.001) was reported in group 2 (3.6g/dL) than in Group 1(2.4g/dL).

In Group 2, 9.7% of the patients (n=9) required a blood transfusion, and 1 patient developed a renal arteriovenous fistula and required embolus therapy. However, in Group 1, only 1.5% of the patients (n=9) suffered a severe hemorrhage and required a blood transfusion postoperatively. In Group 1, a significant decrease in the blood transfusion rate was found compared with that in Group 2 (P=0.039; Table-3).

**Table 3:** Bleeding Complications.

Variable	Group 1	Group 2	P value
Drop in hemoglobin (g/dL) (mean)	2.4	3.6	0.000
Blood transfusion (%)	1.5	9.7	0.039
Arteriovenous fistula(n)	0	1	0.40
Embolus therapy(n)	0	1	0.40

## DISCUSSION

Percutaneous nephrolithotomy is less invasive than open surgery, and is associated with high success rates. Today, percutaneous nephrolithotomy has become an effective and valued procedure for treating kidney and ureter stones. Although percutaneous nephrolithotomy is a minimally invasive technique, it carries out the potential risk of complications.

The complication rates associated with PCNL have been reported to range from 29% to 83% (1–3), and in a recent analysis, the complication rate was 15% (5). Complications related to percutaneous nephrolithotomy include: infection, bleeding, pelvic perforation, urinary fistulas and perforations of adjacent organs. Bleeding is one of the most significant complications.

In the percutaneous nephrolithotomy Global Study analysis, the bleeding complication rate and transfusion rate were 9.4% and 7.0%, respectively (4). In the percutaneous nephrolithotomy study reported by Zehri and coworkers, the overall blood transfusion rate was 14.2% (6). In our study, 9.7% of the patients in Group 2 required a blood transfusion and only 1 patient in Group 1.

The preoperative factors that were reported to be predictors of blood loss in percutaneous nephrolithotomy include age, hypertension, diabetes mellitus (DM), ipsilateral pyelonephritis, the serum creatinine level, stone localization and burden, previous ipsilateral renal stone surgery and extracorporeal shock wave lithotripsy and the degree of hydronephrosis and stone type (7–9). The operative factors were the operation time, puncture calyx and tract number (9). An analysis suggested that BMI may increase the risk of bleeding (10), but other studies reported that BMI was not associated with the complication rates (11–13).

Rosette et al. reported that dilation was a risk factor for bleeding complications, with balloon dilation significantly increasing the risk of bleeding compared with telescopic/serial dilation. In their study, bleeding was reported in 9.4% of the patients with balloon dilation compared with 6.7% with telescopic/serial dilation; the sheath size may have influenced the transfusion rates. The transfusion rate was only 1.1% for the smallest sheath but was 12.0% for the largest sheath (5). We believe that the use of smaller sheaths could help reduce bleeding.

Bleeding is usually caused by a renal vascular injury, which may occur at any step of the percutaneous nephrolithotomy. A renal vascular injury may lead to several consequences, including arteriovenous fistula, pseudoaneurysm, hemorrhage, hypotension, and loss of renal function (14).

Most vascular injuries manifest as hematuria. Bleeding from venous vessels can be managed by simple maneuvers, such as placing the patient in the supine position, positioning a nephrostomy catheter, and clamping the nephrostomy catheter (15).

Arterial lesions may lead to severe acute bleeding. Some types of severe bleeding, such as arteriovenous fistulas and pseudoaneurysms, can be persistent and require specific treatments. The incidence of arteriovenous fistulas or pseudoaneurysms associated with percutaneous nephrolithotomy is approximately 0.8% (16). In Group 2, 1 patient (1.1%) developed a renal arteriovenous fistula. The treatment is more troublesome if the bleeding occurs during the course of the percutaneous nephrolithotomy.

Duplex US and computed tomography angiography are often used to diagnose vascular injuries. Hyperselective renal embolization is considered to be the most appropriate technique for treating renal vascular injuries, with a high success rate and a low complication rate (15). Renal angiography is used to simultaneously diagnose and treat arteriovenous fistula or pseudoaneurysms (17). In our study, the patient who developed a renal arteriovenous fistula was treated with hyperselective renal artery embolus therapy.

Although there are many maneuvers to control bleeding, which may lead the loss of renal function, the prevention of any bleeding should be a major focus during percutaneous nephrolithotomy. To decrease the incidence of hemorrhagic complications, the mini-percutaneous technique has been applied widely. In a recent study, B-mode combined with color Doppler ultrasound guidance in percutaneous nephrolithotomy avoided renal vascular injury, and reduced the risk of bleeding occurrence (18). Penbegül reported that percutaneous nephrolithotomy with ultrasound guidance could be safely performed in children (19). Although the use of ultrasound guiding puncture could avoid vessel lesions, we think it is necessary to select calyces preoperatively.

Although digital subtraction angiography is still regarded as the gold standard to inspect renal vascular anatomy, with the introduction of multi-detector computed tomography, computed tomography angiography is increasingly used to evaluate renal arterial anatomy as it offers an accurate, safe and rapid visualization of the vascular structure (20). Raman et al. reported that the sensitivity of computed tomography angiography for demonstration of the renal artery was 98.5% (21). Computed tomography angiography has the advantage of evaluating not only the main vessels but also renal tumors and stones with only one test (22).

Currently, non-contrast CT has already been shown to be useful in pre-operative planning for percutaneous nephrolithotomy. Although non-contrast CT (Figure-2) may be able to identify the posterior calyx, it cannot display renal vasculature, and reduce vascular complications. In this study, computed tomography angiography was performed preoperatively (Figure-1) and provided a map of the renal vasculature, renal artery, segmental artery and larger vessel branches were displayed accurately in Group 1. By reading the computed tomography angiography, we selected access site(s) far away from larger vessels, and the risk of hemorrhage was reduced. In Group 1, no patient developed a renal arteriovenous fistula and required embolus therapy, and only 1 patient required a blood transfusion.

In this study, there were several limitations. First, the number of enrolled patients was rather small, and further studies are needed to verify our results. Second, there were selection bias in our study due to the retrospective nature. Third, there were some disadvantages of computed tomography angiography, such as the need for intravenous contrast (that affects renal function in patients with renal insufficiency), significant radiation exposure and higher cost.

## CONCLUSIONS

In conclusion, the study showed that patients who underwent computed tomography angiography prior to mini-percutaneous nephrolithotomy had lower drop of hemoglobin and needed less transfusions. The use of computed tomography angiography may be a viable alternative to decrease the likelihood of bleeding complications during mini-percutaneous nephrolithotomy.

## ABBREVIATIONS

CT: = Computed tomography
CTA: = Computed tomography angiography
PCNL: = Percutaneous nephrolithotomy
BMI: = Body mass index
DM: = Diabetes mellitus
